# Preventing long-term disability in CIDP: the role of timely diagnosis and treatment monitoring in a multicenter CIDP cohort

**DOI:** 10.1007/s00415-024-12548-1

**Published:** 2024-07-11

**Authors:** Paula Quint, Christina B. Schroeter, Felix Kohle, Menekse Öztürk, Andreas Meisel, Giuliano Tamburrino, Anne K. Mausberg, Fabian Szepanowski, Ali Maisam Afzali, Katinka Fischer, Christopher Nelke, Saskia Räuber, Jan Voth, Lars Masanneck, Alice Willison, Anna Vogelsang, Bernhard Hemmer, Achim Berthele, Michael Schroeter, Hans-Peter Hartung, Marc Pawlitzki, Stefanie Schreiber, Mark Stettner, Uwe Maus, Sven G. Meuth, Frauke Stascheit, Tobias Ruck

**Affiliations:** 1 Department of Neurology, Medical Faculty and University Hospital Düsseldorf, Heinrich-Heine-University Düsseldorf, Moorenstr. 5, 40225 Duesseldorf, Germany; 2 Department of Neurology, Faculty of Medicine, University of Cologne and University Hospital of Cologne, Cologne, Germany; 3 Department of Neurology and Experimental Neurology, Charité-Universitätsmedizin Berlin, corporate member of Freie Universität Berlin and Humboldt Universität zu Berlin, Berlin, Germany; 4 Neuroscience Clinical Research Center, Charité-Universitätsmedizin Berlin, corporate member of Freie Universität Berlin and Humboldt Universität zu Berlin, Berlin, Germany; 5 Department of Neurology, Essen University Hospital, University Duisburg-Essen, Essen, Germany; 6 Center for Translational Neuro- and Behavioral Sciences (C-TNBS), University of Duisburg-Essen, Essen, Germany; 7 Department of Neurology, Klinikum Rechts der Isar, Technical University Munich School of Medicine and Health, Ismaninger Str. 22, 81675 Munich, Germany; 8 Institute for Experimental Neuroimmunology, Technical University of Munich School of Medicine and Health, Munich, Germany; 9 Munich Cluster for Systems Neurology (SyNergy), 81377 Munich, Germany; 10 Brain and Mind Center, University of Sydney, 94 Mallett St, Sydney, Australia; 11 Department of Neurology, Palacky University Olomouc, Nová Ulice, 779 00 Olomouc, Czech Republic; 12 Department of Neurology, Otto-von-Guericke University, 39120 Magdeburg, Germany; 13 German Center for Neurodegenerative Diseases (DZNE), 39120 Magdeburg, Germany; 14 Center for Behavioral Brain Sciences (CBBS), Otto-von-Guericke University, 39106 Magdeburg, Germany; 15 Department of Orthopaedics and Trauma Surgery, Medical Faculty, Heinrich Heine University Duesseldorf, Moorenstr. 5, 40225 Duesseldorf, Germany

**Keywords:** Neuroimmunology, CIDP, Autoimmune, Therapy

## Abstract

**Background:**

Chronic inflammatory demyelinating polyneuropathy (CIDP) is an inflammatory disease affecting the peripheral nerves and the most frequent autoimmune polyneuropathy. Given the lack of established biomarkers or risk factors for the development of CIDP and patients’ treatment response, this research effort seeks to identify potential clinical factors that may influence disease progression and overall treatment efficacy.

**Methods:**

In this multicenter, retrospective analysis, we have screened 197 CIDP patients who presented to the University Hospitals in Düsseldorf, Berlin, Cologne, Essen, Magdeburg and Munich between 2018 and 2022. We utilized the respective hospital information system and examined baseline data with clinical examination, medical letters, laboratory results, antibody status, nerve conduction studies, imaging and biopsy findings. Aside from clinical baseline data, we analyzed treatment outcomes using the Standard of Care (SOC) definition, as well as a comparison of an early (within the first 12 months after manifestation) versus late (more than 12 months after manifestation) onset of therapy.

**Results:**

In terms of treatment, most patients received intravenous immunoglobulin (56%) or prednisolone (39%) as their first therapy. Patients who started their initial treatment later experienced a worsening disease course, as reflected by a significant deterioration in their Inflammatory Neuropathy Cause and Treatment (INCAT) leg disability score. SOC-refractory patients had worse clinical outcomes than SOC-responders. Associated factors for SOC-refractory status included the presence of fatigue as a symptom and alcohol dependence.

**Conclusion:**

Timely diagnosis, prompt initiation of treatment and careful monitoring of treatment response are essential for the prevention of long-term disability in CIDP and suggest a “hit hard and early” treatment paradigm.

**Supplementary Information:**

The online version contains supplementary material available at 10.1007/s00415-024-12548-1.

## Background and objectives

Chronic inflammatory demyelinating polyneuropathy (CIDP) is an autoimmune disease affecting the peripheral nervous system and the most frequent autoimmune polyneuropathy [1, 2]. The underlying pathophysiological mechanisms are not entirely understood. Immune-mediated processes leading to demyelination and axonal damage of peripheral nerves thought to be important [3, 4]. Recent studies have detected potentially associated IgG4 autoantibodies directed against antigens in nodal and paranodal sections of Ranvier proteins [5–8]. However, the prevalence of these autoantibodies is low [9] and they are increasingly regarded as an independent disease entity [10].

Typical CIDP symptoms are symmetric muscle weakness, sensory disturbances in the limbs and a reduction or loss of deep tendon reflexes [11, 12]. In addition, different clinical presentations must be used to distinguish between several variants of CIDP [11, 13], for which a distinct etiology is being discussed [14, 15]. According to the European Academy of Neurology/Peripheral Nerve Society Guideline (EAN/PNS) 2021, diagnosis of CIDP depends on typical clinical presentation, electrodiagnostic phenotypes and supportive criteria like cerebrospinal fluid (CSF) analysis, imaging, response to treatment and nerve biopsy results [11].

Recommended treatment options in CIDP are either immunoglobulins or corticosteroids [11, 16], which often have to be administered over a period of years or even decades [1]. Plasma exchange can also be used as first therapy but may be associated with severe adverse events and can be a challenge in maintenance therapy due to the risks of central venous access and related coagulopathies [11]. If these therapy regimes fail, off-label therapy with immunosuppressants may be used as a therapeutic option or add-on medication [11], but all with limited evidence.

Previous studies have already identified some biomarkers that correlate with clinical activity [17–21]. However, these findings still have limitations and have not yet been translated into clinical practice [18]. Besides possible associations with other autoimmune diseases, diabetes, hypertension, dietary lifestyle and previous infections [12, 22], the development of CIDP and response to treatment have no clearly recognized risk factors [23, 24]. There is also a lack of objective, validated methods for the serologic measurement of disease activity and treatment response in CIDP patients in clinical practice [25, 26]. Therefore, this study aims to identify potential risk factors that might influence disease progression and overall treatment success.

## Methods

### Study design and cohort

**Fig. 1:**
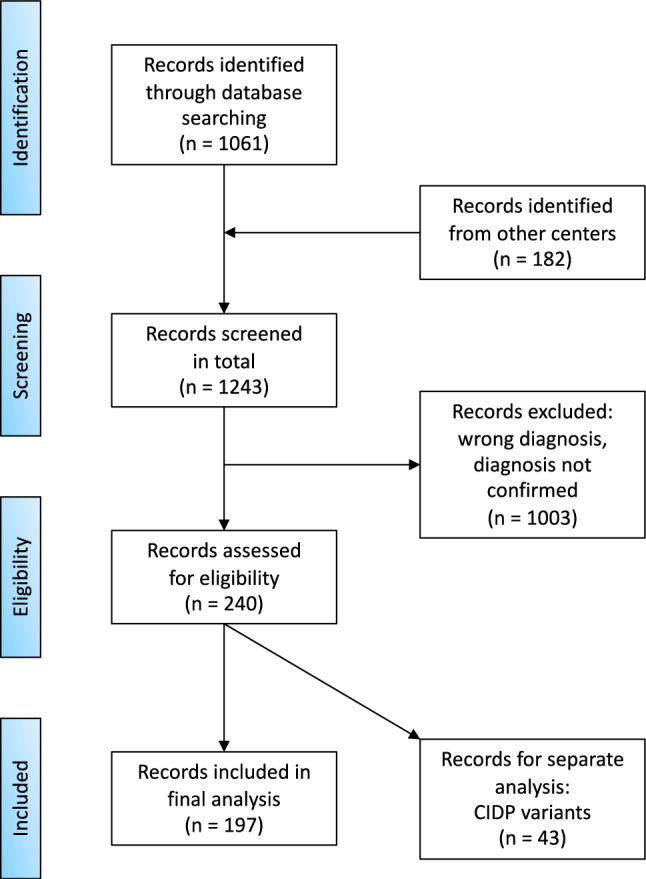
PRISMA flow chart illustrating screening and inclusion of patient records used in this study

In this multicenter, retrospective analysis, we screened patients, who presented with an immune-mediated neuropathy at the University Hospitals in Düsseldorf, Berlin, Cologne, Essen, Magdeburg and Munich (which are all centers in Germany) during the period of 2018–2022. We retrospectively queried the local clinical databases to identify patients with the following International Classification of Diseases (ICD)-10 codes: ICD-10 GM 2022 G60; G61; G62; G63; G64. Outpatient and inpatient hospitalizations were included in this study. Out of 1243 patients that were initially screened, 103 (Düsseldorf), 44 (Berlin), 30 (Cologne), 28 (Munich), 27 (Essen), and 8 (Magdeburg) patients fulfilled the 2021 EAN/PNS criteria from [11] for CIDP. To achieve a more homogeneous cohort, 43 patients diagnosed with a CIDP variant (according to the 2021 EAN/PNS guidelines [11]) were analyzed separately, leaving a total of 197 typical CIDP patients being included in the final analysis (Fig. 1). Baseline data of CIDP variants are shown next to those of typical CIDP patients. In the following analyses only typical CIDP patients were included.

We used the respective hospital information systems to collect the required clinical data from clinical examinations, laboratory tests, nerve conduction studies, imaging studies and nerve biopsies.

Subsequently, the cohort was screened for the following factors: Socio-demographics (sex, age at admission, manifestation and diagnosis), diagnosis (ICD-10 code at admission and discharge, out- or in-patient, time between diagnosis and manifestation, family history), and clinical scores. The latter included the Inflammatory Neuropathy Cause and Treatment (INCAT) arm and leg disability score at the time of first diagnosis and after 12, 24 and 36 months of follow-up, respectively. The INCAT disability score is a widespread rating system to assess disease-related limitation of activity [27]. Further, the Medical Research Council (MRC) score was used to grade the muscle strength of upper arm abductors, elbow flexors, wrist extensors, hip flexors, knee extenders, and foot dorsal flexors on a scale from 0 (plegia) to 5 (full strength). A cumulative MRC sum score, ranging from 0 to 60, was calculated at the time of first diagnosis and after 12, 24, and 36 months [28]. Apart from that, clinical data also included patients’ symptoms and diagnostic data (reflexes; CSF cell number and protein level; findings of neurography, nerve ultrasound and nerve biopsy; serostatus for nodal and paranodal antibodies; levels of creatine kinase (CK), antinuclear antibody (ANA), anti-neutrophil cytoplasmic antibody (ANCA), renal and liver parameters, hemoglobin A1C (HbA1c), Vitamin B12 and folic acid) were included. Additionally, data was supplemented by comorbidities and therapy details (time to first treatment, first therapy, response to first therapy, adverse drug reactions (ADR), switch to second therapy, all further therapies). Finally, we analyzed the outcomes of the enrolled patients by comparing and correlating their initiation of therapy (early vs. late initiation of first therapy) with treatment response (responders vs. refractory patients), looking for potential risk factors.

### Definitions

To define patients who responded to therapy and those who did not, we used the recommendations of the 2021 EAN/PNS guideline for objective therapy response [11] and the definition of treatment response and non-response already used in previously published studies by Allen et al. [25]*.* and Wieske et al. [17]. We than adapted the Standard of Care (SOC) definition, which was already employed in an on-going phase 2 trial of a complement component C1 directed monoclonal antibody SAR445088 (ClinicalTrials.gov identifier (NCT number): NCT04658472):

*SOC-responder* (original: “SOC-treated”): Objective response to first therapy, defined by at least one of the following points: ≥ one point decrease in adjusted INCAT score, ≥ four points increase in Rasch-built Overall Disability Scale (RODS) total score, ≥ three points increase in MRC Sum score, ≥ eight kPa improvement in mean grip strength (in one hand), or an equivalent improvement based on documented information.

*SOC-refractory* Evidence of failure or inadequate response to first therapy, defined by at least one of the following points: Persistent INCAT score ≥ two after treatment for a minimum of 12 weeks, ≥ one point decrease in adjusted INCAT score, increase in RODS total score ≥ four points, increase in MRC Sum score ≥ three, mean grip strength improvement of ≥ eight kilopascals (one hand), or equivalent lack of improvement based on information from medical records.

Additionally, we expanded this definition and considered the time point of 12 months after the start of first therapy to define:

*Sustained SOC-responder:* Patients who remained responsive to treatment 12 months after starting therapy and thus still fulfilled the SOC responder criteria. The latter are defined as an objective treatment response according to the above-mentioned criteria.

*Transitioned SOC-refractory* Patients who stopped responding to treatment 12 months after starting treatment and therefore switched to the SOC-refractory group. Transitioned SOC-refractory patients have an objective failure to treatment according to the above criteria.

### Ethics

This study was approved by the ethics committees of the Heinrich Heine University Duesseldorf (registration number 2022-1809), the Charité Berlin (no. EA4/166/23), Cologne (21-1079), Essen University Hospital (no. 18–8084-BO and 21-9930-BO), Technical University Munich (approval number 2022-204-S) and University Hospital Magdeburg (no. 07/17 and 07/17 2023). Data was anonymized before statistical analysis.

### Statistical analysis

Statistical analysis was performed using GraphPad Prism 9.5^®^ (GraphPad Software, Inc., San Diego, CA, USA). The cohort data were depicted as means including standard error of the mean (SEM) or absolute (n) and relative frequencies (%). To analyze further questions related to the cohort, the following methods were used: Non-parametric tests were used in most cases because the values were not normally distributed. For comparison of two independent groups, Mann–Whitney-U-test was applied. For comparison of paired groups, Wilcoxon signed-rank test was applied. In case of normal distributed data, Welch-test was used. To test multiple hypotheses, two-way ANOVA or Kruskal–Wallis test was used. Fisher’s exact test was used to investigate the correlation between the categorical variables. In addition, a multiple logistic regression was used, and Odds ratios were calculated. A p-value < 0.05 was set as statistically significant. Additionally, an Alluvial plot was created using the free website https://www.rawgraphs.io.

## Results

### Baseline characteristics

The clinical and demographic baseline characteristics of Typical CIDP patients versus CIDP variants are presented in Table 1. Overall, 197 typical CIDP patients were screened, of whom 136 (69%) were male and 110 (56%) were treated in an outpatient department. Out of 43 CIDP variant patients, 31 (72%) were male and 30 (70%) were outpatients. CIDP variant patients were older than typical CIDP patients at first manifestation (58 ± 2 versus 57 ± 1 years) and diagnosis (60 ± 2 versus 59 ± 1 years), with a longer time between manifestation and diagnosis (30 ± 3 versus 19 ± 2 months). The most common CIDP variants were multifocal (20 patients, 47%) and distal (11 patients, 26%) CIDP.

**Table 1:** Clinical and demographic baseline characteristics of CIDP patients

Characteristic	Typical CIDP	CIDP variants
Total	197	43
Sex (n (%))		
Male	136 (69)	31 (72)
Female	61 (31)	12 (28)
Age (mean ± SEM)		
Age at study begin (years)	69 ± 7	63 ± 3
Age at first manifestation (years)	57 ± 1	58 ± 2
Age at diagnosis (years)	59 ± 1	60 ± 2
Time between manifestation and diagnosis (months)	19 ± 2	30 ± 3
Type of consultation (n (%))		
Outpatient	110 (56)	30 (70)
In-patient	59 (30)	10 (23)
No data available	28 (14)	3 (7)
CIDP variant^a^ (n (%))		
Distal CIDP		11 (26)
Multifocal CIDP		20 (47)
Focal CIDP		0 (0)
Motor CIDP		3 (7)
Sensory CIDP		9 (21)

Individual clinical and demographic data of CIDP patients, subdivided into typical CIDP and CIDP variants. Results are shown as absolute values including their relative percentages or as mean ± SEM

*CIDP* chronic inflammatory demyelinating polyneuropathy, *SEM* standard error of the mean

^a^According to EAN/PNS guidelines from 2021 [11]

Common comorbidities in typical CIDP patients were cardiovascular (104 patients, 54%) and metabolic disorders (61 patients, 32%). Diabetes mellitus type 2 was a comorbidity in 43 patients (22%) (Supplementary Table 1).

Details on diagnostic data are presented in Table 2. In both typical CIDP and CIDP variants, most patients (72% in typical and 60% in CIDP variants) exhibited areflexia in at least one muscle tendon reflex. The analysis of cerebrospinal fluid showed a normal mean cell number in both groups (reference range: < 5 cells/µl) with higher protein levels of 94 ± 7 mg/dl (reference range: 20–50 mg/dl) in typical CIDP patients. In addition, these patients more often exhibited an albuminocytological dissociation (116 patients, 72% of all patients who underwent a lumbar puncture). In the evaluation of neurography at baseline, most patients (62% of typical CIDP and 65% of CIDP variants) displayed a combined axonal-demyelinating damage. The motor nerve conduction criteria according to the EAN/PNS guidelines were fulfilled in 178 (92%) typical CIDP and 35 (81%) CIDP variant patients, respectively. A biopsy of N. suralis was available in 71 typical CIDP patients, of which 66 (93%) were pathologic. In CIDP variants, 22 (88%) of 25 available biopsies were pathologic. Histomorphological characteristics of an exemplary sural nerve biopsies are shown in Supplementary Fig. 1, which were required to show loss of myelinated fibers, focal accumulation of macrophages and T cells in the endoneurium and evidence of frank demyelination/hypomyelination and remyelination on teased fibers.

Mean blood values are summarized in Table 2. The mean HbA1c was 5.8 ± 0.1% in typical CIDP and 5.7 ± 0.1% in CIDP variant patients (reference range: 4.8–5.7%). The mean CK level was slightly elevated both in typical CIDP (180 ± 14 U/l) and CIDP variants (191 ± 24 U/l) (reference range: < 171 U/l). 17 (9%) typical CIDP and three (7%) CIDP variant patients had elevated ANA and two (1%)/one (2%) patients had elevated ANCA antibodies, respectively. The serostatus of antibodies was tested in 104 typical CIDP and three CIDP variant patients, and all of them were tested negative. Thus, the serostatus was not included in further analyses.

**Table 2:** Diagnostic data of CIDP patients

Characteristic	Typical CIDP	CIDP variants
Reflexes (n (%))		
Areflexia	142 (72)	26 (60)
Hyporeflexia	51 (26)	6 (14)
Normal reflexes	0 (0)	8 (19)
Hyperreflexia	0 (0)	0 (0)
Not available	4 (2)	3 (7)
Cerebrospinal fluid		
CSF cell number (mean ± SEM in cell/µl)	4 ± 1	2 ± 0.2
CSF protein level (mean ± SEM in mg/dl)	94 ± 7	67 ± 10
Albuminocytological dissociation (n (%))	116 (79)	16 (60)
Not available	36 (18)	16 (37)
Neurography (n (%))		
Demyelinating	68 (35)	11 (26)
Axonal	6 (3)	2 (5)
Axonal-demyelinating	120 (62)	28 (65)
Not available	3 (2)	2 (5)
Motor nerve conduction criteria^a^		
In > 2 nerves	178 (92)	35 (81)
In 1 nerve	13 (7)	5 (12)
None	0	0
Not available	6 (3)	3 (7)
Sensory nerve conduction criteria^a^		
In > 2 nerves	156 (81)	26 (60)
In 1 nerve	34 (18)	9 (21)
None	0	5 (12)
Not available	7 (4)	3 (7)
Biopsy of N. suralis (n (%))		
Not available	126 (65)	18 (42)
Pathologic^a^	66 (34)	22 (51)
Normal	5 (3)	3 (7)
Blood values		
HbA1c (mean ± SEM in %)	5.8 ± 0.1	5.7 ± 0.1
CK level (mean ± SEM in U/l)	180 ± 14	191 ± 24
ANA elevated (n (%))	17 (9)	3 (7)
ANCA elevated (n (%))	2 (1)	1 (2)
Renal parameters elevated (n (%))	23 (12)	3 (7)
Liver parameters elevated (n (%))	23 (12)	7 (16)

Acquired clinical and diagnostic data of CIDP patients, subdivided into typical CIDP and CIDP variants. Results are shown as absolute values including their relative percentages or as mean ± SEM

*ANA* antinuclear antibody, *ANCA* anti-neutrophil cytoplasmatic antibody, *CIDP* chronic inflammatory demyelinating polyneuropathy, *CK* creatine kinase, *CSF* cerebrospinal fluid, *HbA1c* hemoglobin A1C, *SEM* standard error of the mean

^a^According to EAN/PNS guidelines form 2021 [11]

To sum up, CIDP variant patients presented with higher age and a longer delay between symptom onset and diagnosis compared to typical CIDP patients, while both groups displayed similar neurological impairments and pathological findings. However, typical CIDP patients had higher cerebrospinal protein levels and were more likely to have albuminocytological dissociation.

### Symptoms and scores at initial admission

**Fig. 2:**
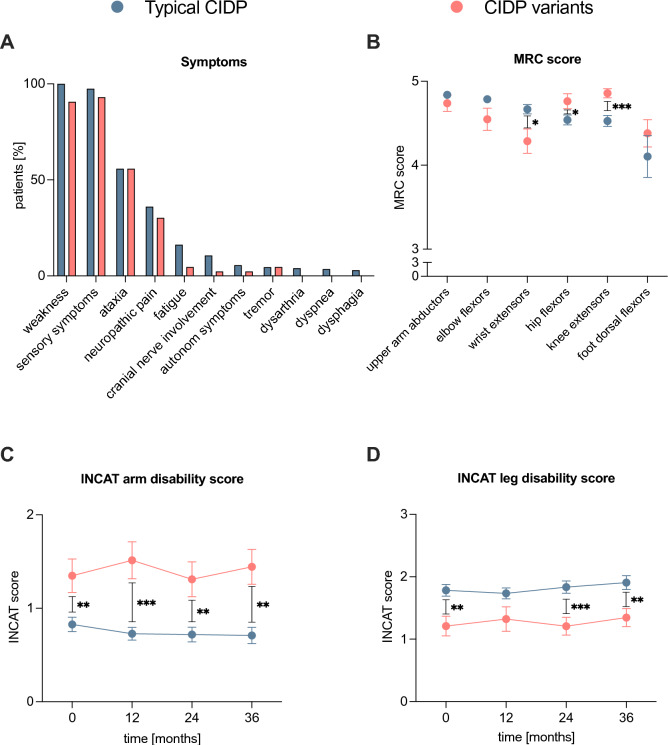
Clinical symptoms and disease course of typical CIDP versus CIDP variants. **A**, **B** Clinical symptoms and MRC scores on initial admission are depicted. Symptoms are displayed as relative percentage of all patients. **C**, **D** INCAT scores were determined at initial diagnosis (0), after 12, 24 and 36 months. Mean scores ± SEM are shown. Data was available from (typical CIDP/CIDP variants) 180/43 (diagnosis), 151/35 (after 12 months), 121/29 (after 24 months) and 110/27 (after 36 months) patients. A *p*-value ≥ 0.05 was classified as not significant, *p* < 0.05 (*) as significant, *p* < 0.01 (**), *p* < 0.001 (***), and *p* < 0.0001 (****) as highly significant. *CIDP* chronic inflammatory demyelinating polyneuropathy, *INCAT* inflammatory neuropathy cause and treatment, *MRC* Medical Research Council, *SEM* standard error of the mean

At initial admission, the most common symptoms were weakness (100% of typical CIDP, 91% of CIDP variants), sensory disturbances (97% of typical CIDP, 93% of CIDP variants), and/or ataxia (both 56%). (Fig. 2A). Mean upper extremity MRC scores at baseline were higher in typical CIDP patients with a significant (p < 0.05) difference in wrist extensors, while the mean MRC scores of the lower extremity were higher in CIDP variants with a significant difference in hip flexors (p < 0.05) and knee extensors (p < 0.001). (Fig. 2B). The INCAT arm disability score of CIDP variants remained significantly (p < 0.01 and p < 0.001) higher than in the typical CIDP patients at all time points whereas the INCAT leg disability score of typical CIDP patients was significantly higher at diagnosis (p < 0.01), 24 (p < 0.001), and 36 (p < 0.01) months (Fig. 2C, D).

Overall, typical CIDP patients displayed significantly worse INCAT and MRC scores in the lower extremity, while CIDP variant patients had worse scores in the upper extremity.

### Choice of first treatment affects the probability of SOC-responder status and treatment change

**Fig. 3:**
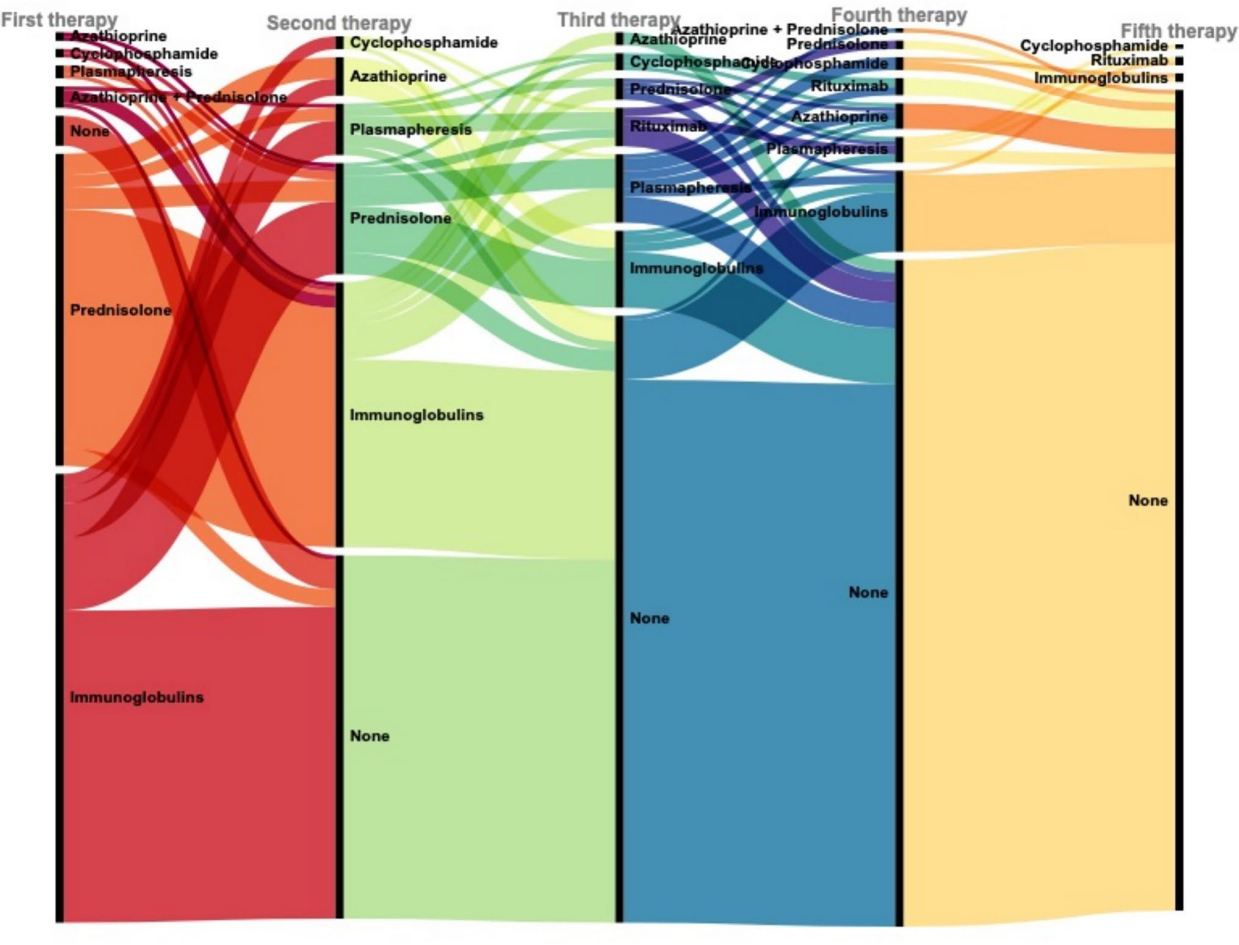
First to fifth therapy of typical CIDP patients. Alluvial plot that shows the individual therapies of CIDP patients, chronologically from first to fifth. In this context, none means no change of therapy. *CIDP* chronic inflammatory demyelinating polyneuropathy

For a general overview of therapies, see Fig. 3**,** which shows the first to fifth successive therapies performed. Out of 187 patients that received immunomodulatory therapy, 122 (65%) changed to a second therapy. While most patients receiving immunoglobulins did not need to switch their first therapy (59%), most patients (88%) who received prednisolone as their first therapy switched to a second therapy.

Baseline data of patients who received immunoglobulins as first therapy compared to those who received prednisolone can be found in Supplementary Table 2. Immunoglobulin-treated patients were more likely to be male, had a shorter time from first manifestation to diagnosis, and had worse INCAT arm and leg disability and MRC sum scores at diagnosis.

**Fig. 4:**
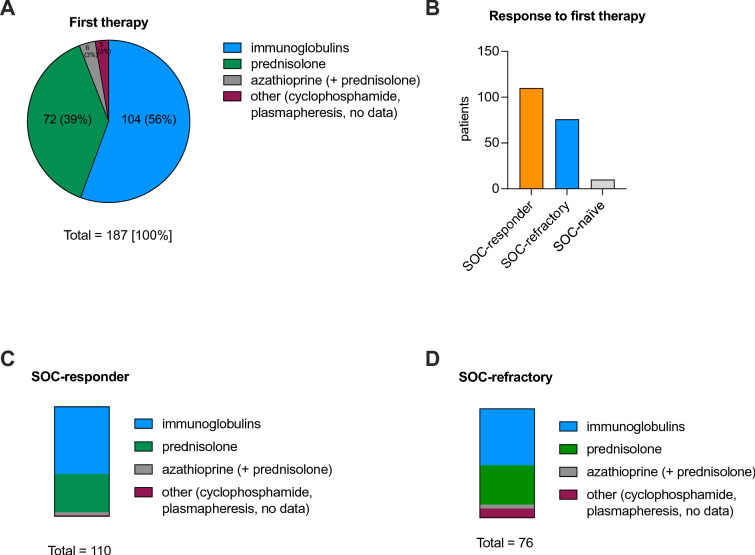
Details on first treatment regimen of typical CIDP patients. **A** First treatment regimen of all patients in % (n = 187). The absolute values are indicated next to their corresponding relative percentages. **B** Patients’ response to their first therapies according to the SOC criteria are illustrated. **C** Fractions of the respective treatment regimens in absolute values of all SOC-responder patients (n = 110). **D** Distribution of the respective treatment regimens in absolute values of all SOC-refractory patients (n = 76). *CIDP* chronic inflammatory demyelinating polyneuropathy, *SOC* standard of care

Figure 4 displays details on the first therapy regimens of the typical CIDP cohort. As first treatment, most patients received intravenous immunoglobulins (56%) or prednisolone (39%), either continuously or recurrently (Fig. 4A). Overall, 110 (56%) patients responded to their first therapy and were thus assigned to the status SOC-responder (Fig. 4B). 76 (39%) patients did not respond to their first therapy and were assigned to the status SOC-refractory (Fig. 4B). The majority of SOC-responders received immunoglobulins (67 patients, 61%) or prednisolone (38 patients, 35%) as their initial therapy (Fig. 4C). 35 patients of the SOC-refractory cohort (46%) received immunoglobulins and 32 (42%) prednisolone (Fig. 4D). 10 patients did not receive immunomodulatory therapy and were therefore not included in the analysis (“SOC-naïve”). One patient received immunomodulatory therapy, but data on treatment response were missing (Fig. 4B).

In summary, most patients received intravenous immunoglobulins or prednisolone as initial treatment and the majority responded to their first therapy.

### Patients with a late start of first therapy had worse clinical scores than those with early treatment onsets

**Fig. 5:**
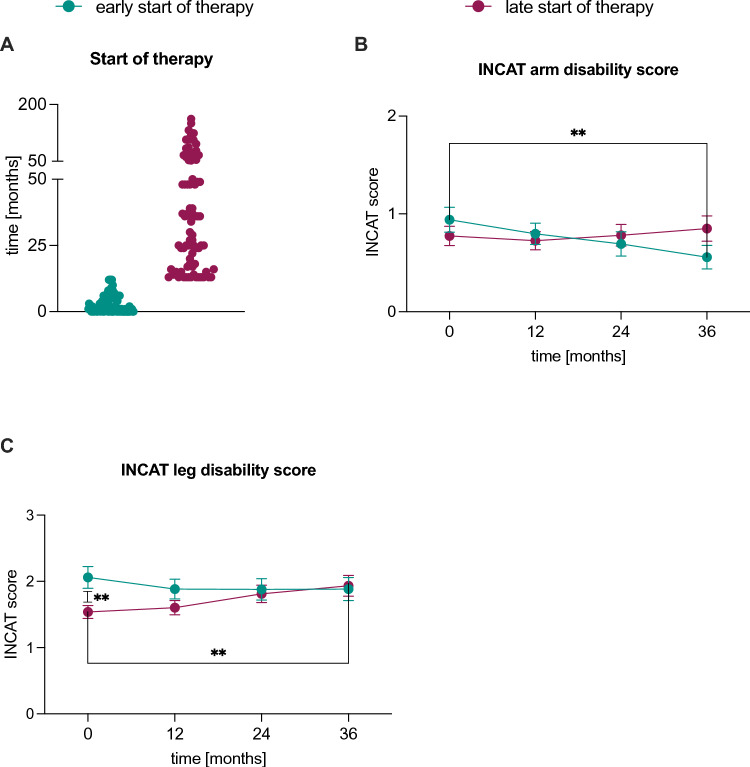
Comparison of early versus late onset of therapy in typical CIDP patients. **A** The start of the first therapy after first symptom manifestation is depicted in months: early onset of therapy was defined as start of first therapy up to 12 months after first manifestation of symptoms. Late start of therapy was set as start of first therapy more than 12 months after first manifestation of symptoms. **B**, **C** Mean INCAT arm and leg disability scores ± SEM at diagnosis (0), after 12, 24 and 36 months, respectively. Data was available from (early/late start of therapy) 85/80 (at diagnosis), 60/73 (after 12 months), 49/64 (after 24 months) and 43/59 (after 36 months) patients. A *p*-value ≥ 0.05 was classified as not significant, *p* < 0.05 (*) as significant, *p* < 0.01 (**), *p* < 0.001 (***), and *p* < 0.0001 (****) as highly significant. For a better clarity, not all significant results are shown. *CIDP* chronic inflammatory demyelinating polyneuropathy, *INCAT* inflammatory neuropathy cause and treatment, *SEM* standard error of the mean

For a more in-depth examination of treatment outcomes in CIDP patients, we conducted a comparative analysis between those who initiated treatment early (defined as commencing therapy within 12 months of their initial symptom manifestation) and those with a late onset of therapy (more than 12 months after their first symptoms appeared) as illustrated in Fig. 5. The INCAT arm disability scores (Fig. 5B) of patients with an early onset showed a significant (p < 0.01) decrease 36 months after diagnosis, as well as 24 months after diagnosis (p < 0.05), between 12 and 24 months (p < 0.05), 12 and 36 months (p < 0.001) and between 24 and 36 months (p < 0.01) after diagnosis. The INCAT leg disability scores (Fig. 5C) of patients with a late treatment start was significantly (p < 0.01) lower at diagnosis and showed a significant increase 24 (p < 0.05) and 36 (p < 0.01) months after diagnosis, as well as between 12 and 24 (p < 0.05) and between 12 and 36 (p < 0.01) months after diagnosis.

Hence, our data showed a significant worsening of INCAT leg disability scores within the time frame of 36 months in patients who received a late start of therapy and an improvement in INCAT arm disability score in patients who received therapy early.

### SOC-refractory status is associated with several factors and worse clinical outcomes

**Fig. 6:**
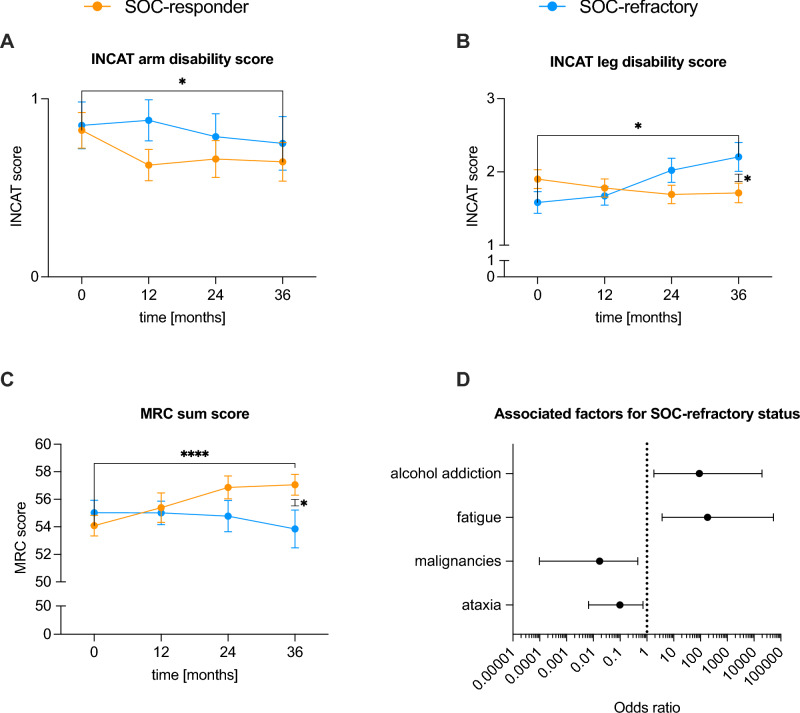
Comparison of treatment strategies, scores, and associated factors of SOC-responder versus SOC-refractory patients in typical CIDP patients. **A**, **B** Mean INCAT arm and leg disability scores ± SEM at diagnosis (0 months), after 12, 24 and 36 months, respectively. Data was available from (SOC-responder/SOC-refractory) 102/67 (at diagnosis), 86/58 (after 12 months), 68/47 (after 24 months) and 65/40 (after 36 months) patients. **C** Mean MRC sum scores ± SEM at diagnosis (0 months), after 12, 24 and 36 months, respectively. Data was available from (SOC-responder/SOC-refractory) 108/74 (at diagnosis), 48/48 (after 12 months), 37/37 (after 24 months) and 35/34 (after 36 months) patients. **D** Associated factors for a SOC-refractory status. Values are presented as Odds ratios with a 95% confidence interval. A *p*-value ≥ 0.05 was classified as not significant, *p* < 0.05 (*) as significant, *p* < 0.01 (**), *p* < 0.001 (***), and *p* < 0.0001 (****) as highly significant. For a better clarity, not all significant results are indicated. *CIDP* chronic inflammatory demyelinating polyneuropathy, *INCAT* inflammatory neuropathy cause and treatment, *MRC* Medical Research Council, *SEM* standard error of the mean, *SOC* standard of care

The INCAT arm disability scores of SOC-responder patients showed a significant decrease 12 (p < 0.001), 24 (p < 0.01) and 36 (p < 0.05) months after diagnosis (Fig. 6A). In contrast, the INCAT leg disability score of SOC-refractory patients significantly increased 24 and 36 months (p < 0.05) after diagnosis, as well as between 12 and 24 months (p < 0.01), 24 and 36 months (p < 0.01) and between 24 and 36 months (p < 0.05) after diagnosis. Also, the INCAT leg disability score of SOC-responder patients 36 months after diagnosis was significantly (p < 0.05) lower than in the SOC-refractory group (Fig. 6B). Mean MRC sum scores of SOC-responder patients significantly (p < 0.0001) improved 36 months after diagnosis and were significantly higher than in the SOC-refractory group at this time point. In addition, the SOC-responder patients showed a significant improvement 24 months after diagnosis (p < 0.001), as well as between 12 and 24 months (p < 0.01) and between 24 and 36 months (p < 0.001) after diagnosis. In contrast, SOC-refractory patients showed a significant (p < 0.05) deterioration between 24 and 36 months after diagnosis (Fig. 6C**).**

Factors associated with SOC-refractory status are shown in Fig. 6D. Significant factors identified by multiple logistic regression were alcohol dependence and subjective fatigue as a symptom. Malignancies and ataxia were less often found in SOC-refractory patients.

In summary, patients with a SOC-refractory status showed a worsening of their clinical scores associated with several factors.

### Sustained SOC-responder patients showed clinical improvement over time

**Fig. 7:**
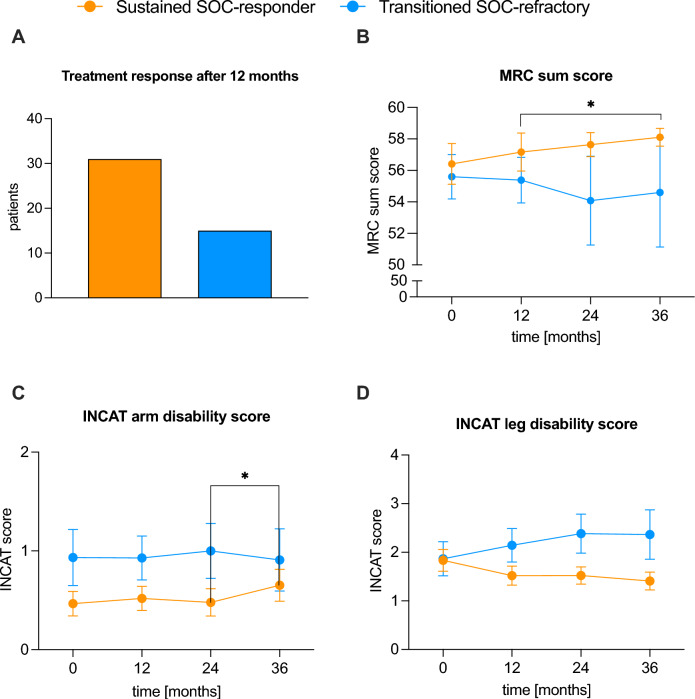
Overview and clinical scores of sustained SOC-responders and transitioned SOC-refractory patients in typical CIDP patients. **A** The total amount of patients with the status sustained SOC-responder (patients who still met the SOC-responder status at 12 months follow-up) and sustained SOC-refractory (patients who transitioned to a SOC-refractory status at 12 months follow-up) after 12 months, respectively. **B** Mean MRC sum scores ± SEM of these patient subgroups after 12, 24 and 36 months, respectively. Data was available from (sustained SOC-responder/transitioned SOC-refractory) 29/15 (at diagnosis), 24/13 (after 12 months), 22/12 (after 24 months) and 20/10 (after 36 months) patients. **C**, **D** Mean INCAT arm and leg disability scores ± SEM of the depicted treatment-response cohorts after 12, 24 and 36 months, respectively. Data was available from (sustained SOC-responder/transitioned SOC-refractory) 30/15 (at diagnosis), 27/14 (after 12 months), 23/13 (after 24 months) and 23/11 (after 36 months) patients. *CIDP* chronic inflammatory demyelinating polyneuropathy, *INCAT* inflammatory neuropathy cause and treatment, *MRC* Medical Research Council, *SEM* standard error of the mean, *SOC* standard of care

Lastly, we evaluated the clinical scores of SOC-responder patients from the Düsseldorf cohort, who still showed an improvement of clinical scores after 12 months of follow-up (“sustained SOC-responders”) and those who showed a deterioration after 12 months (“transitioned SOC-refractory”) (Fig. 7). Out of 110 patients, who had a SOC-responder status at the start of their first therapy, 31 patients were sustained SOC-responders. Meanwhile, 15 patients switched to a SOC-refractory status (transitioned SOC-refractory) (Fig. 7A). The MRC sum score of sustained SOC-responder patients was higher at all time points (0, 12, 24, and 36 months after diagnosis) than for the transitioned SOC-refractory cohort, with a significant (p < 0.05) improvement between 12 and 36 months after diagnosis (Fig. 7B). Both INCAT arm and leg disability scores of sustained SOC-responder patients were lower at all time points. However, differences were not significant except for an (p < 0.05) improvement in the INCAT arm disability score of sustained SOC-responder patients between 24 and 36 months after diagnosis (Fig. 7C, D). Similar to the comparison of the SOC-responder versus -refractory group, favoring factors for a sustained SOC-responder status were investigated using multiple logistic regression. However, none of the tested clinical parameters showed a significant impact on the therapy outcome after 12 months of follow-up.

In conclusion, sustained SOC-responder patients showed an improvement in clinical scores over time, whereas transitioned SOC-refractory patients deteriorated. Potential risk factors investigated for a sustained SOC-responder status did not show significant effects on therapy outcomes after 12 months of follow-up.

## Discussion

CIDP is associated with a significant burden of disease, with many people experiencing severe limitations in activities of daily living [29]. As there are no robust serologic biomarkers or risk factors for the development of CIDP or treatment response [23, 24], the aim of this study was to gain a better understanding of disease progression and potentially modulating factors.

In the current cohort of CIDP patients, the majority (95%) received an immunomodulatory therapy, of which 94% comprised a recommended first line therapy according to the EAN/PNS guidelines (immunoglobulins, corticosteroids or plasma exchange) [11]. Nevertheless, in our study only 56% showed an objective response to their first therapy, which is consistent with previously published studies [17, 30]. 65% of the cohort at hand switched to a second therapy, which is a higher rate than seen in previous studies [30]. The reasons for a change of therapy included existing side effects (such as rash, headache or flu-like symptoms) or the potential high risk of side effects from long-term steroid therapy.

Although the retrospective design of this study, the uneven distribution of patients between the different centers and the selection bias for the choice of first treatment are limitations, the clinical and demographic baseline characteristics of our cohort are consistent with those that have been reported in other studies: the mean age at first manifestation of symptoms and diagnosis was between 40 and 60 years [31] and men were more often affected [32]. Besides, sensory symptoms, muscle weakness and areflexia were common symptoms [12]. We compared typical CIDP patients with CIDP variants and found a clinically more prominent involvement of the upper extremity in CIDP variants. Laboratory findings included elevated CK levels (180 ± 14 in typical CIDP and 191 ± 24 U/l in CIDP variants), which has previously been described in the literature [33]. However, as the sample size was too small and we wanted to achieve as homogeneous a cohort as possible, we did not include the CIDP variants in the more detailed analyses.

The mean time from first symptoms to diagnosis in typical CIDP patients was 19 ± 2 months. Comorbidities such as diabetes mellitus type 2 were present in 22% and malignancies in 19% of patients, highlighting the challenge of diagnosing CIDP [24] as these diseases may be an alternate potential cause of polyneuropathy and thus delay the diagnosis of CIDP. However, an early diagnosis and start of therapy is crucial to prevent long-lasting disability and nerve damage [16, 34, 35]. This was supported by our observation that patients with a late start of therapy showed a significant deterioration in INCAT leg disability scores at follow-up. Hence, an early diagnosis using the current electrophysiological and supportive criteria published by the EAN/PNS [11] may beneficially influence disease progression.

Analysis of treatment response demonstrated that SOC-refractory patients suffered from a significant worsening of their INCAT score over time, characterizing them as clinically more impaired. Although a deterioration of the INCAT score within the first eight weeks after treatment onset is part of the definition of this status, the scores of SOC-refractory patients worsened beyond this interval, suggesting that an early and detailed evaluation of the treatment response and, consecutively, an adjustment of the therapy regime, is of importance for the course of disease. Associated factors with a SOC-refractory disease course were alcohol addiction and fatigue. Fatigue as a non-specific symptom in CIDP that has been described more frequently in recent years and has been associated with increased disability and poorer quality of life [36]. However, we were unable to identify distinct clinical factors or biomarkers that predict an unfavorable therapy outcome. Specifically, the therapy regimen, socio-economic data, disease progression, and diagnostic blood and cerebrospinal fluid values did not influence the therapy outcome in CIDP patients. This could be explained by the limited number of patients included in our study and the uneven distribution among centers. A center effect with sicker patients could also have an impact on the results.

Of note, we extended our view and examined whether SOC-responder patients were able to maintain their status for 12 months or transitioned to a refractory status. Here, we found that regular monitoring of patients’ treatment response and early treatment changes in case of insufficient treatment response is crucial in clinical practice. Otherwise, a slow clinical deterioration during disease progression may remain unnoticed.

In summary, our research highlights the urgent need for advances in the understanding of CIDP, including its risk factors, pathophysiology and therapeutic approaches, and describes the current knowledge gaps that require further investigation and research. We focused on the clinical deterioration of CIDP patients by extending the definition of SOC-responder patients to sustained SOC-responders or transitioned SOC-refractory, respectively. Regular monitoring of treatment response should be integrated more frequently into clinical routine in order to allow treatment changes in time. Additionally, we could point out the importance of an early diagnosis and start of treatment to halt lasting disability favoring a hit hard and early treatment strategy. However, the complexity of clinical management of CIDP remains as the lack of reliable biomarkers capable of indicating clinical disease activity and identifying patients at risk of disease worsening continues to impede the integration of effective clinical practice. Hence, there is an urgent need for prospective clinical and molecular tools to advance the diagnosis and management of CIDP.

## Supplementary Information

Below is the link to the electronic supplementary material.

Supplementary file1 (PDF 1018 kb)

## Data Availability

All data sets generated and analyzed during this current study and statistical analysis are available from the corresponding author upon reasonable request.

## Abbreviations

ADR: Adverse drug reaction
ANA: Antinuclear antibody
ANCA: Anti-neutrophil cytoplasmic antibody
ANOVA: Analysis of variance
CASPR: Contactin-associated protein
CIDP: Chronic inflammatory demyelinating polyneuropathy
CK: Creatin kinase
CNTN: Contactin
CSF: Cerebrospinal fluid
DADS: Distal acquired demyelinating symmetric polyneuropathy
EAN/PNS: European Academy of Neurology/Peripheral Nerve Society
HbA1c: Hemoglobin A1C
ICD: International Classification of Diseases
INCAT: Inflammatory neuropathy cause and treatment
M.: Morbus
MADSAM: Multifocal acquired demyelinating sensory and motor neuropathy
MGUS: Monoclonal gammopathy of undetermined significance
MRC: Medical Research Council
N.: Nervus
NF: Neurofascin
ODSS: Overall Disability Sum Score
RODS: Rasch-built Overall Disability Scale
SEM: Standard error of the mean
SOC: Standard of care
