# Supplementary material for: Preventing long-term disability in CIDP: the role of timely diagnosis and treatment monitoring in a multicenter CIDP cohort
Source: J Neurol. 2024 Jul 11;271(9):5930–43. doi: 10.1007/s00415-024-12548-1 (PMC11377626; doi:10.1007/s00415-024-12548-1)
Supplement: Supplementary file 1 — Supplementary file1 (PDF 1018 kb) [file 415_2024_12548_MOESM1_ESM.pdf]

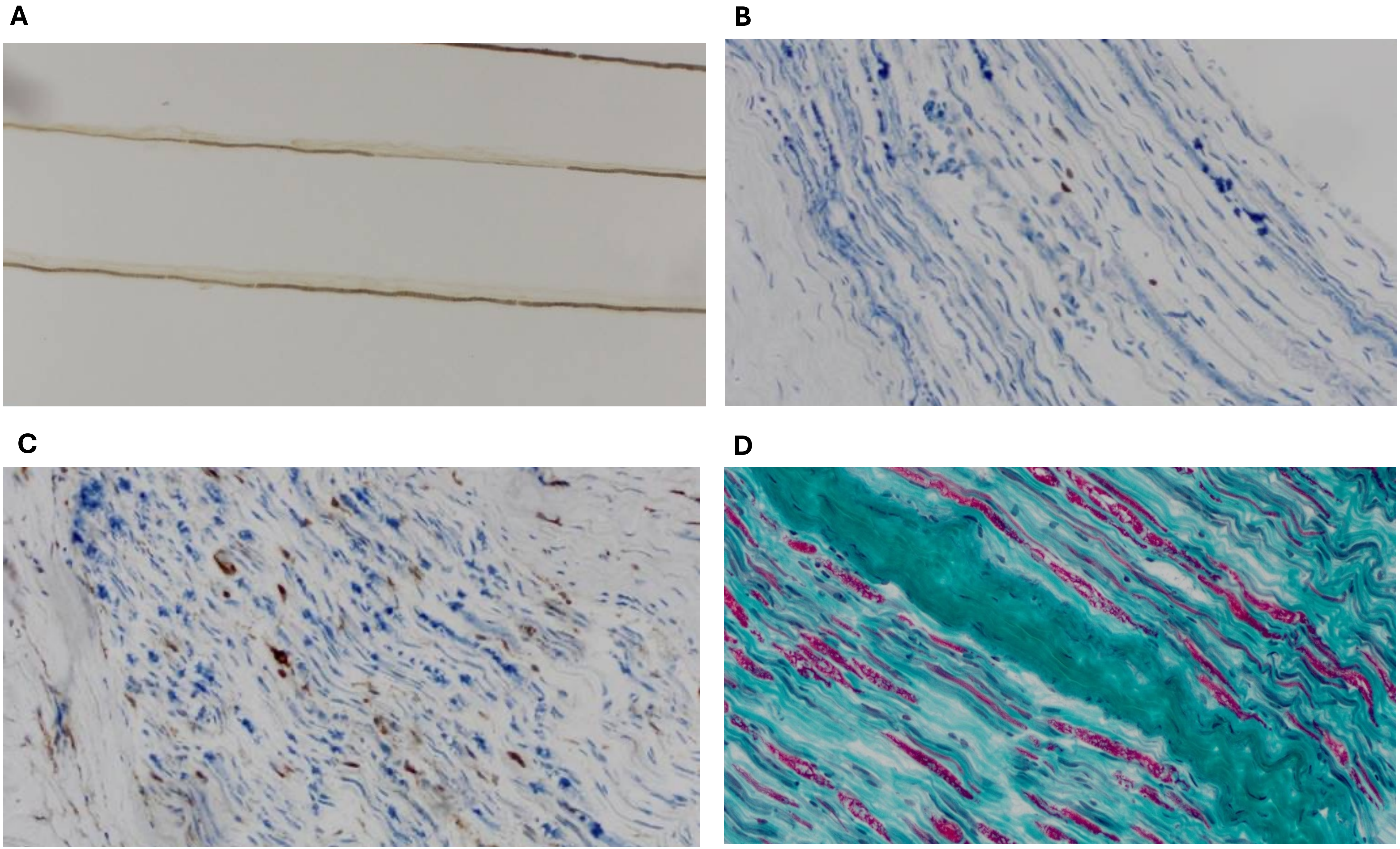
A
B
C
D

Comorbidity	*n*	*%*
Other neuropathies	7	4
Guillain-Barré syndrome	7	
Autoimmune diseases	26	13
Hypothyroidism	14	
Rheumatoid arthritis	3	
Cardiovascular diseases	104	54
Arterial hypertension	87	
Coronary heart disease	19	
Atrial fibrillation	17	
Pulmonary diseases	28	15
Chronic obstructive pulmonary disease	9	
Sleep apnea	8	
Bronchial asthma	5	
Neurological diseases	40	21
Stroke	9	
Restless legs syndrome	3	
Parkinson's disease	3	
Psychiatric diseases	27	14
Depression	22	
Anxiety disorder	3	
Malignancies	36	19
Monoclonal gammopathy of undetermined significance	21	
Prostate cancer	5	
Multiple myeloma	3	
Colon cancer	3	
Ophthalmological diseases	15	8
Cataract	11	
Glaucoma	3	
Metabolic diseases	61	32
Diabetes mellitus type 2	43	
Hypercholesterolemia	19	
Obesity	6	
Gastroenterological diseases	20	10
Diverticulitis	5	
Steatosis hepatis	4	
Gastritis	3	
Renal diseases	20	10
Chronic renal failure	13	
Diabetic nephropathy	2	
Infectious diseases	8	4
Lyme disease	4	
Human immunodeficiency virus	3	
Urinary tract infection	2	
Other diseases	49	25
Disc prolapse	11	
Prostatic hyperplasia	5	
Arthrosis	4	

Supplementary Table 1 Prevalence of comorbidities among typical CIDP patients

Individual comorbidities of CIDP patients. Results are shown as total values (n) or absolute frequencies (%).

Abbreviations: CIDP, chronic inflammatory demyelinating polyneuropathy.

Characteristic	Immunoglobulins	Prednisolone
**Total**	**105**	**72**
**Sex *(n (%))***		
Male	78 (74)	45 (63)
Female	27 (26)	27 (37)
**Age *(mean ± SEM)***		
Age at study begin (years)	62 ± 2	62 ± 2
Age at first manifestation (years)	58 ± 1	57 ± 1
Age at diagnosis (years)	59 ± 1	59 ± 2
Time between manifestation and diagnosis (months)	17 ± 3	21 ± 4
**Type of consultation *(n (%))***		
Outpatient	56 (53)	42 (58)
In-patient	30 (29)	22 (31)
**Clinical scores *(mean ± SEM)***		
INCAT arm disability score at diagnosis	0.9 ± 0.1	0.6 ± 0.1
INCAT leg disability score at diagnosis	2 ± 0.1	1.3 ± 0.01
MRC sum score at diagnosis	53.5 ± 0.9	56.1 ± 0.6
**Therapy**		
Time to first treatment from diagnosis in months *(mean ± SEM)*	4.4 ± 0.8	4.7 ± 1.4
SOC-responder *(n (%))*	67 (64)	39 (54)
SOC-refractory *(n (%))*	36 (35)	33 (46)
Switch to second therapy *(n (%))*	43 (41)	63 (88)

Supplementary Table 2 Baseline data of first therapies of typical CIDP patients

Baseline data of typical CIDP patients receiving immunoglobulins as first therapy versus prednisolone. Results are shown as total values (n) with absolute frequencies (%) or mean ± SEM.

Abbreviations: CIDP, chronic inflammatory demyelinating polyneuropathy; INCAT, Inflammatory Neuropathy Cause and Treatment; MRC, Medical Research Council; SEM, standard error of the mean; SOC, standard of care
